# Endoscopic ultrasound-guided antegrade stenting with a long duodenal extension using an ultra-flexible spiral-covered stent

**DOI:** 10.1055/a-2893-7177

**Published:** 2026-06-24

**Authors:** Koichiro Mandai, Takato Inoue

**Affiliations:** 1 Department of Gastroenterology 38334 Kyoto Second Red Cross HospitalKyotoJapan

**Fig. 1:**
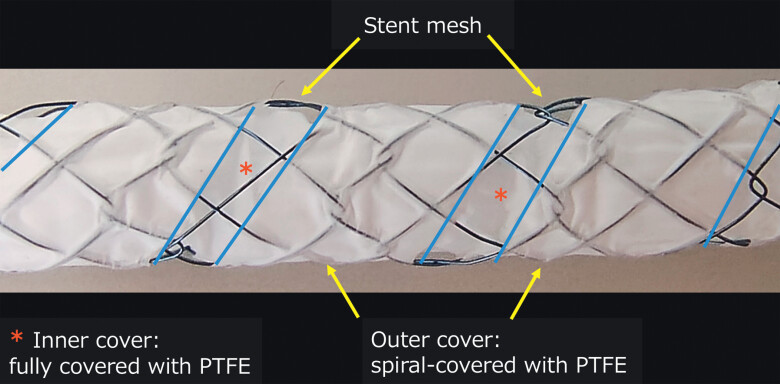
The stent consists of a fully PTFE-covered inner layer and a spiral PTFE-covered outer layer.

**Fig. 2:**
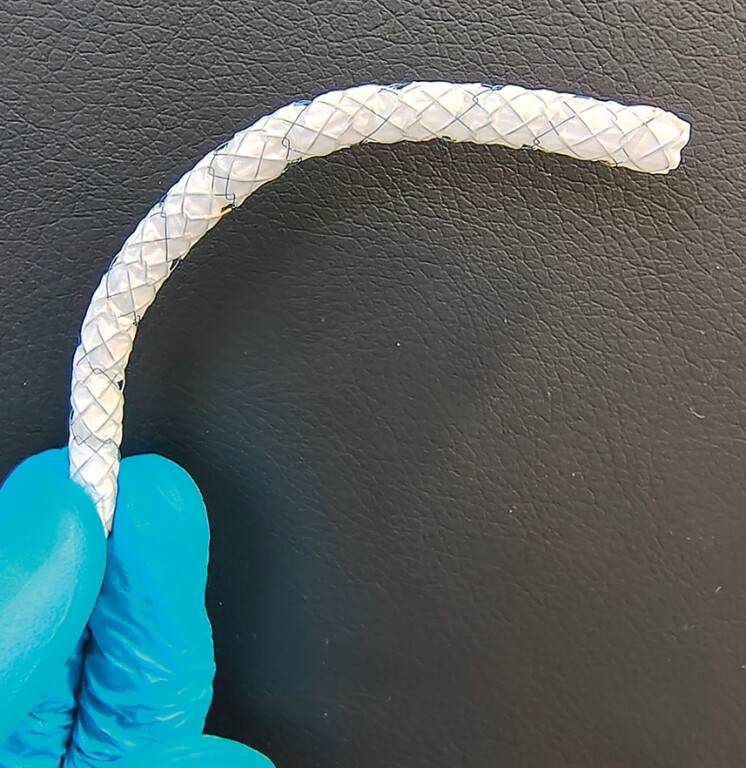
The novel stent is ultra-flexible and exhibits low axial force, allowing it to conform smoothly to curved anatomical structures.

Duodenal invasion is a risk factor for early biliary stent dysfunction in patients with pancreatic cancer. 1​ In such patients, stent placement with a long duodenal extension may prevent early dysfunction caused by reflux cholangitis and food impaction. 2​ ​3​ Recently, a novel biliary stent has become available in Japan, which has several features that may be advantageous for endoscopic ultrasound-guided antegrade stenting (EUS-AG) with a long duodenal extension. The belt-like gaps in the spiral outer cover reduce stent migration while maintaining drainage of the cystic duct and pancreatic duct, thereby potentially reducing the risks of cholecystitis and pancreatitis ( Fig. 1 ). The 6-mm stent diameter also reduces these risks. 4​ The slim 6.5-Fr delivery system enables insertion without dilation of the puncture tract, and its ultra-flexible design improves the safety of the long duodenal extension ( Fig. 2 ).

**Fig. 3:**
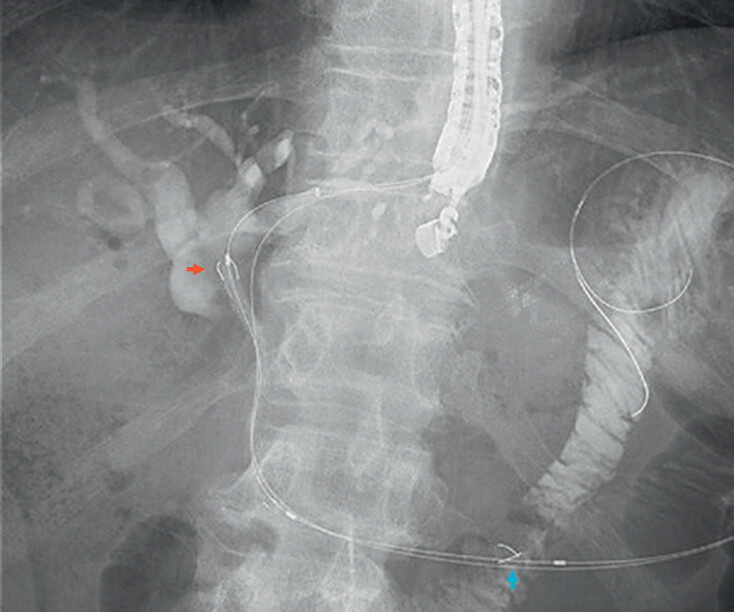
The novel stent (6 mm×12 cm) was successfully deployed across the biliary stricture and papilla, extending from the bile duct (red arrow) to the third portion of the duodenum (blue arrow).

**Fig. 4:**
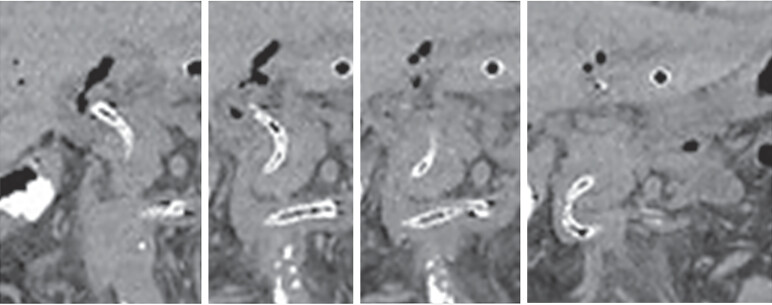
Computed tomography performed the following day demonstrated that the novel stent conformed smoothly to the curvature of the bile duct and duodenum, reflecting its ultra-flexibility.

A 79-year-old man with unresectable pancreatic cancer developed obstructive jaundice following gastrointestinal bypass surgery for malignant duodenal stenosis. Therefore, EUS-guided hepaticogastrostomy with antegrade stenting was performed. After the intrahepatic bile duct was punctured using a 19G needle, a 0.025-inch guidewire was advanced. Subsequently, two guidewires were inserted across the biliary stricture using an uneven double-lumen cannula and advanced into the third part of the duodenum. 5​ The novel stent (6 mm×12 cm) was successfully deployed across the stricture and papilla with a long duodenal extension ( Fig. 3 ). Finally, a fully covered self-expandable metal stent was placed from the left intrahepatic bile duct into the stomach. The next day, computed tomography demonstrated that the antegrade stent conformed to the curvature of the bile duct and duodenum, reflecting its ultra-flexibility ( Fig. 4 and Video 1 ). The patient's jaundice resolved, and he was discharged without any procedure-related adverse events.

**Video 1** EUS-guided antegrade stenting with a long duodenal extension using a novel ultra-flexible spiral-covered stent with a 6.5-Fr slim delivery system.


This novel biliary stent may facilitate safer and more effective EUS-AG with long duodenal extension.

Endoscopy_UCTN_Code_TTT_1AR_2AL
